# miR-7-5p Promotes Hepatic Stellate Cell Activation by Targeting Fibroblast Growth Factor Receptor 4

**DOI:** 10.1155/2020/5346573

**Published:** 2020-06-10

**Authors:** Shuxia Tian, Min Chen, Bing Wang, Yonglong Han, Haonan Shang, Junming Chen

**Affiliations:** ^1^Shanghai University of Medicine & Health Sciences Affiliated Zhoupu Hospital, China; ^2^Shanghai Jiao Tong University Affiliated Sixth People's Hospital, China

## Abstract

**Aims:**

Fibroblast growth factor receptor 4 (FGFR4) is a key mediator that protects the liver from chronic injury. MicroRNA-7 (miR-7) is a tumor suppressor and associated with lipid homeostasis in the liver. This study was designed to examine the role of the miR-7-5p/FGFR4 axis in liver fibrogenesis.

**Methods:**

TargetScan was employed to predict microRNAs that targeted FGFR4 on the 3′-untranslated region (3′-UTR). miR-7-5p and FGFR4 expression in pathological liver tissues and LX-2 cells was determined using qRT-PCR and an immunoblotting assay. A dual-luciferase assay was conducted to validate the target prediction. A Cell Counting Lit-8 assay was performed to assess the proliferation ability of LX-2 cells. Hydroxyproline content in LX-2 cells was measured using a hydroxyproline assay. The expression of hepatic stellate cell (HSC) activation markers was examined using qRT-PCR and an immunoblotting assay.

**Results:**

FGFR4 was a putative target of miR-7-5p. In LX-2 cells, miR-7-5p targeted FGFR4 by binding to 3′-UTR. FGFR4 was downregulated, but miR-7-5p was markedly enhanced in the liver samples as the degree of liver fibrosis rose. miR-7-5p was negatively associated with FGFR4 expression in liver tissues. The miR-7-5p inhibitor blocked the lipopolysaccharide-induced proliferation and activation of LX-2 cells, and FGFR4 overexpression inhibited LX-2 cell proliferation and activation triggered by miR-7-5p.

**Conclusion:**

miR-7-5p promotes HSC proliferation and activation by downregulating FGFR4.

## 1. Introduction

Most types of chronic liver diseases lead to the accumulation of extracellular matrix proteins, such as collagen, which is the cause of liver fibrosis [1]. Activation of hepatic stellate cells (HSCs) is one step toward liver fibrosis. In a normal liver, HSCs are quiescent; however, in an injured liver, they are activated and transdifferentiate into myofibroblastic HSCs, which are identified as the major collagen-producing cells [2]. Fibroblast growth factor receptor 4 (FGFR4) is a fibroblast growth factor (FGF) receptor activated by endocrine FGFs. In liver cells, FGFR4 activation by FGF19 restrains the gluconeogenesis and stimulates the synthesis of glycogen and protein [3]. In clinical trials, liver fibrosis in nonalcoholic steatohepatitis patients was attenuated by FGF19 analogue treatment [4–6]. Recent evidence indicates that FGFR4 functions as an important mediator of homeostasis in the liver [7]. Deletion of FGFR4 and Fgf15 (murine orthologue of FGF19) leads to significant liver fibrosis compared with little mates [8], suggesting that the FGFR4/FGF19 axis has antifibrotic properties.

MicroRNAs (miRNAs) can bind to the mRNA 3′-untranslated region (3′-UTR) to contribute to the posttranscriptional gene regulation [9]. A growing number of evidences suggest that miRNAs play essential roles in various human diseases, including liver diseases [10]. It has been documented that miRNAs can modulate cell survival and proliferation, inflammation, and glucose and lipid metabolism in the liver [11]. Accumulating evidences suggested that dysregulation of miRNAs is involved in the process of liver fibrosis as well as HSC activation [12, 13], such as miR-29 families [14], miR-199, miR-200 [15], and miR-34 [16]. Since miRNAs can be easily quantified in various body fluids [17–19], they have great potential as biomarkers and therapeutic agents for human diseases.

At the present study, we aimed to characterize miRNAs modulating FGFR4/FGF19 signaling to get to know the molecular mechanism of liver fibrogenesis. Our findings indicated a novel role for miR-7-5p in the modulation of FGFR4/FGF19 signaling.

## 2. Materials and Methods

### 2.1. Cell Culture and Transfection

LX-2 cells were provided by the Cell Bank of Type Culture Collection of the Chinese Academy of Sciences (Shanghai, China). Cells were grown in RPMI 1640 medium (HyClone, Logan, UT, USA) containing 1% penicillin-streptomycin solution (Solarbio, Beijing, China) and 10% fetal bovine serum (FBS; Invitrogen, Carlsbad, CA, USA) in a humidified 37°C incubator with 5% CO_2_. The negative control and miR-7-5p mimic and inhibitor were obtained from GenePharma (Shanghai, China). For investigating the effect of miR-7-5p on the expression of FGFR4, the scramble-miR control (NC), miR-7-5p mimic, or miR-7-5p inhibitor was transfected into LX-2 cells using Lipofectamine 2000 (Invitrogen, Carlsbad, CA, USA). To evaluate the effect of FGFR4 on the behavior of miR-7-5p-overexpressing LX-2 cells, cells treated with the miR-7-5p mimic were transfected with a blank pcDNA3.1(+) vector (Vector, Addgene, Watertown, MA, USA) or a pcDNA3.1-FGFR4 expression vector (oeFGFR4) using Lipofectamine 2000. After 48 h cell transfection, gene expression on mRNA and protein levels was detected. The cells were treated with lipopolysaccharides (LPS) (Desite, Chengdu, China) at 0, 50, 100, or 200 ng/ml for the indicated time as shown in figures. The same volume of solvent was used as a vehicle control.

### 2.2. Liver Specimens

Fifteen patients with mild liver fibrosis (F1), 15 patients with severe fibrosis (F2-F3), and 15 patients with cirrhosis (F4-F5) were enrolled in this study. Liver biopsy tissue from patients was scored for fibrosis according to the METAVIR system [20]. Liver samples were resected from patients and scored at -80°C until use. This study was approved by the ethics committee of Shanghai University of Medicine & Health Sciences Affiliated Zhoupu Hospital, and written informed consent was obtained from all patients.

### 2.3. Target Prediction

The potential miRNAs that could target FGFR4 were predicted using TargetScan 7.1 (http://www.targetscan.org/) [21].

### 2.4. RNA Extraction and Quantitative Real-Time PCR (qRT-PCR)

TRIzol (Invitrogen, Carlsbad, CA, USA) was used to extract total RNA from LX-2 cells. Total RNA was converted to cDNA using the RevertAid First Strand cDNA Synthesis Kit (Thermo Fisher Scientific, Waltham, MA, USA). Following this, cDNA was quantified using the ABI 7300 real-time PCR system (Applied Biosystem, Foster City, CA, USA) with the SYBR Green/ROX qPCR Mix (Thermo Fisher Scientific). The internal reference for mRNA expression was glyceraldehyde-3-phosphate dehydrogenase (GAPDH) and Homo sapiens RNA, U6 small nuclear 1 (RNU6-1) for miRNA expression. The primers are listed in Table 1.

### 2.5. Immunoblotting

RIPA buffer (Beyotime Bio., Shanghai, China) was used to prepare cell extracts for immunoblotting. Afterwards, total protein content was determined using a Bio-Rad protein assay kit (Bio-Rad Laboratories, Hercules, CA, USA). Protein samples were electroblotted onto polyvinylidene difluoride (PVDF) membranes. TBST (Tris-buffered saline containing Tween 20) buffer with 5% bovine serum albumin was used to block the membranes. The membranes were blocked at room temperature for 1 h, and then, the corresponding primary antibodies, *α*-SMA (Affinity Biosciences, Cincinnati, OH, USA; AF1032; 1 : 500), COL1A1 (Affinity Biosciences; AF7001; 1 : 500), TGF-*β* (Abcam, Cambridge, MA, USA; Ab179695; 1 : 3000), FGFR4 (Affinity Biosciences; DF10316; 1 : 1000), and GAPDH (Cell Signaling Technology, Danvers, MA, USA; #5174; 1 : 2000), in TBST buffer were used to incubate the membranes. The membranes were kept at 4°C overnight. On the next day, the membranes were incubated with secondary antibodies (Beyotime) at room temperature for 1 h. An enhanced chemiluminescence chromogenic substrate (Thermo Fisher Scientific) was used for visualized protein bands.

### 2.6. Dual-Luciferase Assay

FGFR4 3′-UTR was amplified and subcloned into a psiCHECK-2 vector (Promega, Madison, WI, USA), and the constructed plasmid was named wt-FGFR4-3UTR. Site-directed PCR was employed with a high-fidelity enzyme (TaKaRa, Shiga, Japan) to produce the mutated FGFR4 3′-UTR as described before [22], and the constructed plasmid was named mut-FGFR4-3UTR. Lipofectamine 2000 was used to cotransfect wt/mut-FGFR4-3UT and NC or the miR-7-5p mimic into LX-2 cells, respectively. The Dual-Luciferase Reporter Assay System (Promega, Madison, WI, USA) was employed to assess the firefly and Renilla luciferase activity 48 h after transfection.

### 2.7. Cell Proliferation Assay

Cells with different treatments were inoculated to each well of 96-well plates and grown in a 37°C humidified atmosphere for 12, 24, or 48 h. A Cell Counting Kit-8 (CCK-8, SAB, Nanjing, China) assay was performed according to the manufacturer's instructions. All experiments were conducted at least three times.

### 2.8. Measurement of Hydroxyproline Content

Hydroxyproline content in liver cells was measured using a hydroxyproline assay kit (Jiancheng, Nanjing, China).

### 2.9. Statistical Analysis

Data were displayed as the mean ± standard deviation. GraphPad Prism (San Diego, CA, USA) was employed for statistical analyses. Differences between groups were evaluated using the *t*-test. The Pearson correlation coefficient was calculated to measure the strength of a linear association between the mRNA level of miR-7-5p and FGFR4 in liver tissue samples. *P* < 0.05 was regarded as statistically significant.

## 3. Results

### 3.1. miR-7-5p Negatively Regulated FGFR4 in HSCs

**Figure 1:**
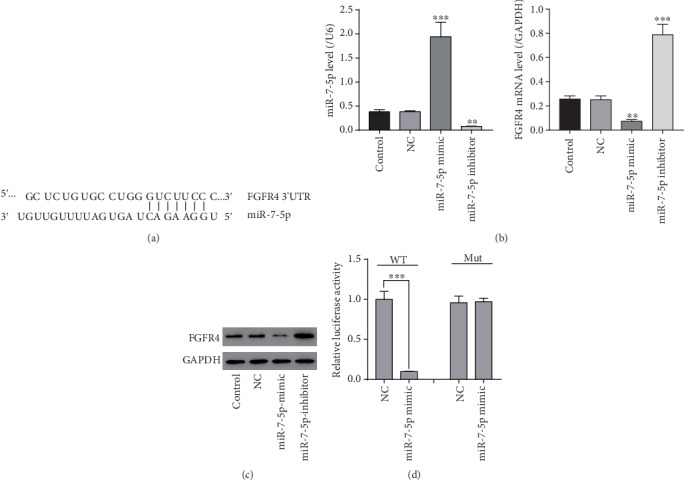
FGFR4 is targeted by miR-7-5p in LX-2 cells. (a) The miR-7-5p potential binding sites on FGFR4. (b) mRNA expression of miR-7-5p and FGFR4 in LX-2 cells transfected with the NC and miR-7-5p mimic or inhibitor. (c) Protein expression of FGFR4 in LX-2 cells transfected with the NC and miR-7-5p mimic or inhibitor. (d) The relative luciferase activity in LX-2 cells detected by the dual-luciferase reporter assay. ^∗∗^*P* < 0.01, ^∗∗∗^*P* < 0.001 versus NC.

*In silico* analysis revealed that FGFR4 was a putative target of miR-7-5p (Figure 1(a)). To examine the impact of miR-7-5p on the expression of FGFR4 in HSCs, the miR-7-5p mimic or inhibitor was transfected into LX-2 cells. NC was used as a control. miR-7-5p expression was significantly enhanced in miR-7-5p-overexpressing cells but suppressed in cells treated with the miR-7-5p inhibitor compared to the controls (Figure 1(b)). FGFR4 was markedly decreased in miR-7-5p-overexpressing LX-2 cells, while it was greatly increased in miR-7-5p inhibitor-treated HSCs, compared to NC and control groups (Figures 1(b) and 1(c)), suggesting that miR-7-5p negatively modulated the expression of FGFR4 in HSCs.

To check if miR-7-5p can bind to FGFR4 3′-UTR directly, wt-FGFR4-3UTR and mut-FGFR4-3UTR luciferase reporter plasmids were constructed, and the dual-luciferase reporter assay was conducted in LX-2 cells. The relative luciferase activity in miR-7-5p-overexpressing cells treated with wt-FGFR4-3UTR was significantly reduced compared to the control group, while no significant change was seen in luciferase activity in miR-7-5p-overexpressing cells treated with mut-FGFR4-3UTR compared with the corresponding controls (Figure 1(d)). Our results implied that miR-7-5p targeted FGFR4 through its 3′-UTR.

### 3.2. miR-7-5p Negatively Regulated FGFR4 in Pathological Liver Tissues

**Figure 2:**
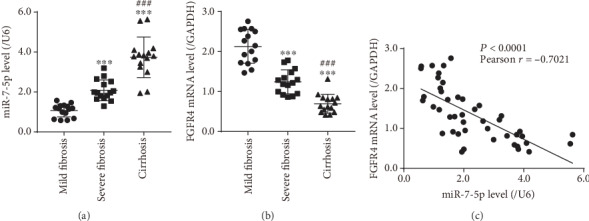
The expression of miR-7-5p and FGFR4 in liver tissues with different degrees of fibrosis. (a) miR-7-5p level in liver tissues with different degrees of fibrosis. (b) The expression of FGFR4 in liver tissues with different degrees of fibrosis. (c) Correlation between miR-7-5p and FGFR4 expression in pathological liver tissues. ^∗∗∗^*P* < 0.001 versus mild fibrosis, ^###^*P* < 0.001 versus severe fibrosis.

miR-7-5p levels in 15 mild fibrosis samples, 15 severe fibrosis samples, and 15 cirrhosis samples were determined using qRT-PCR. miR-7-5p was sharply raised in severe fibrosis or cirrhosis samples compared to mild fibrosis (Figure 2(a)). Compared with severe fibrosis samples, miR-7-5p was significantly upregulated in cirrhosis. On the contrary, FGFR4 was markedly reduced in severe fibrosis or cirrhosis samples compared to mild fibrosis (Figure 2(b)). Compared with severe fibrosis samples, FGFR4 expression was significantly dropped in cirrhosis. As shown in Figure 2(c), miR-7-5p was inversely correlated with FGFR4 level in liver tissues from patients with varying extent of liver fibrosis (*r* = 0.7021, *P* < 0.0001).

### 3.3. LPS Promoted miR-7-5p Expression and Suppressed FGFR4 Expression in HSCs

**Figure 3:**
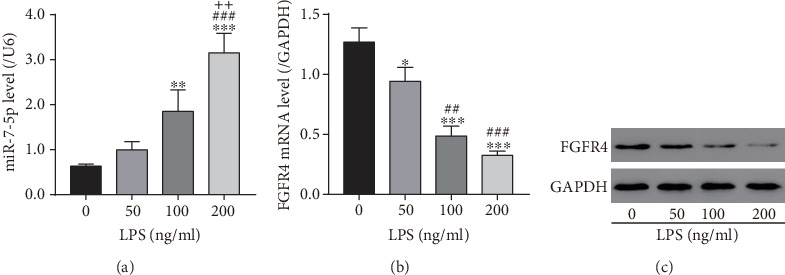
Lipopolysaccharide treatment induced miR-7-5p but inhibited FGFR4 expression in LX-2 cells. LX-2 cells were treated with different concentrations of lipopolysaccharides as indicated. (a) miR-7-5p levels in LX-2 cells measured by qRT-PCR. (b) FGFR4 mRNA levels in LX-2 cells measured by qRT-PCR. (c) FGFR4 protein levels in LX-2 cells measured by western blot. ^∗^*P* < 0.05, ^∗∗^*P* < 0.01, and ^∗∗∗^*P* < 0.001 versus 0 ng/ml; ^##^*P* < 0.01, ^###^*P* < 0.001 versus 50 ng/ml; ^++^*P* < 0.01 versus 100 ng/ml.

LPS increased the production of miR-7-5p but inhibited FGFR4 mRNA and protein expression in a dose-dependent manner (Figure 3), suggesting that LPS mediated the miR-7-5p/FGFR4 module in HSCs.

### 3.4. miR-7-5p Inhibitor Blocked LPS-Induced HSC Proliferation and Activation

**Figure 4:**
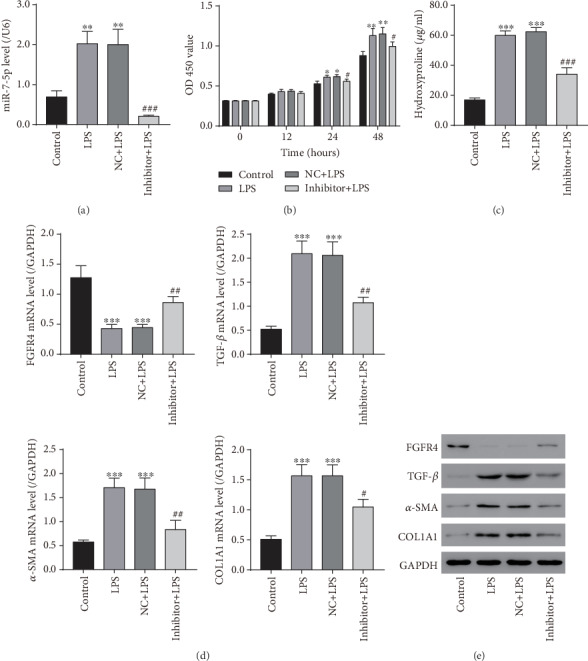
The miR-7-5p inhibitor blocked LPS-induced LX-2 cell proliferation and activation. LX-2 cells were treated with the NC or miR-7-5p inhibitor with or without 100 ng/ml LPS. (a) miR-7-5p levels in LX-2 cells measured by qRT-PCR. (b) OD 450 was measured for 0, 12, 24, and 48 hours later with CCK-8 reagents. (c) The levels of hydroxyproline content in LX-2 cells measured with a hydroxyproline assay kit. (d) mRNA levels of FGFR4, TGF-*β*1, *α*-SMA, and COL1A1. (e) Protein levels of FGFR4, TGF-*β*1, *α*-SMA, and COL1A1 measured by western blot. LPS: lipopolysaccharides; NC: scramble-miR control. ^∗^*P* < 0.05, ^∗∗^*P* < 0.01, and ^∗∗∗^*P* < 0.001 versus control; ^#^*P* < 0.05, ^##^*P* < 0.01, and ^###^*P* < 0.001 versus NC+LPS.

The miR-7-5p inhibitor was introduced to inhibit miR-7-5p production in HSCs. Under the condition of 100 ng/ml LPS, the miR-7-5p inhibitor or NC was transfected into LX-2 cells. miR-7-5p was markedly raised in the LPS group compared to the vehicle control, while it was markedly reduced in LX-2 cells treated with the miR-7-5p inhibitor and 100 ng/ml LPS compared to LX-2 cells treated with NC and 100 ng/ml LPS (Figure 4(a)). Next, we examined the proliferation activity of LX-2 cells treated with the miR-7-5p inhibitor using the CCK-8 assay. As presented in Figure 4(b), LX-2 cell proliferation capability was significantly promoted when treated only with 100 ng/mL LPS or transfected with NC and treated with LPS compared with the vehicle control. In contrast, when treated with the miR-7-5p inhibitor and LPS, LX-2 cell proliferation capability was markedly repressed compared to LX-2 cells treated with NC and LPS. These data implied that the miR-7-5p inhibitor blocked LX-2 cell proliferation induced by LPS. We then measured the hydroxyproline content (a marker for HSC activation) and expression of FGFR4 and three HSC activation markers, TGFB1, *α*-SMA, and COL1A1, in LX-2 cells. As shown in Figures 4(c)–4(e), LPS treatment greatly increased the hydroxyproline content and expression levels of markers in LX-2 cells compared to the vehicle control; however, miR-7-5p inhibitor treatment greatly reduced the elevation of hydroxyproline content and marker levels induced by LPS. As the target of miR-7-5p, FGFR4 was significantly downregulated in the LPS group compared to the vehicle control, while it was markedly increased in LX-2 cells treated with the miR-7-5p inhibitor and LPS compared to those treated with NC and LPS (Figures 4(d) and 4(e)). The data suggested that the miR-7-5p inhibitor can effectively block HSC activation.

### 3.5. FGFR4 Overexpression Inhibited miR-7-5p-Promoted HSC Proliferation and Activation

**Figure 5:**
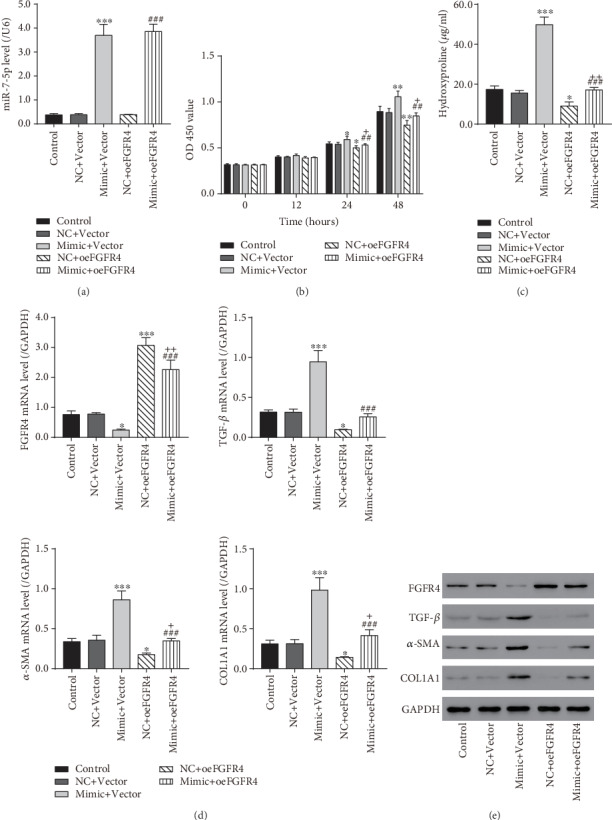
FGFR4 overexpression inhibited the proliferation and activation of LX-2 cells triggered by miR-7-5p. LX-2 cells were cotransfected with the NC or miR-7-5p mimic and empty vector or FGFR4 overexpression vector. (a) miR-7-5p levels in LX-2 cells were measured by qRT-PCR. (b) OD 450 was measured for 0, 12, 24, and 48 hours later with CCK-8 reagents. (c) The levels of hydroxyproline content in LX-2 cells measured with a hydroxyproline assay kit. (d) mRNA levels of FGFR4, TGF-*β*1, *α*-SMA, and COL1A1 measured by qRT-PCR. (e) Protein levels of FGFR4, TGF-*β*1, *α*-SMA, and COL1A1 measured by western blot. NC: scramble-miR control; Vector: empty vector; oeFGFR4: FGFR4 overexpression vector. ^∗^*P* < 0.05, ^∗∗∗^*P* < 0.001 versus NC+Vector; ^##^*P* < 0.01, ^###^*P* < 0.001 versus mimic+Vector; ^+^*P* < 0.05, ^++^*P* < 0.01 versus NC+oeFGFR4.

To verify that FGFR4 was a downstream effector regulated by miR-7-5p in liver fibrosis, the NC/miR-7-5p mimic and pcDNA3.1 empty vector or pcDNA3.1-FGFR4 expression vector were cotransfected into LX-2 cells. miR-7-5p was markedly increased in LX-2 cells treated with the miR-7-5p mimic and pcDNA3.1 empty vector compared to those treated with NC, and the pcDNA3.1 empty vector, miR-7-5p mimic, and pcDNA3.1-FGFR4 expression vector treatment further upregulated miR-7-5p in LX-2 cells compared to LX-2 cells treated with the miR-7-5p mimic and pcDNA3.1 empty vector (Figure 5(a)). The subsequent CCK-8 assay demonstrated that LX-2 cell proliferation was greatly enhanced in the mimic+Vector group but markedly inhibited in the NC+oeFGFR4 group 24 h after transfection compared to the NC+Vector group (Figure 5(b)). The miR-7-5p mimic and pcDNA3.1-FGFR4 expression vector treatment greatly inhibited LX-2 cell proliferation compared with the mimic+Vector group but significantly promoted LX-2 cell proliferation compared with the NC+oeFGFR4 group. These data indicated that FGFR4 overexpression blocked LX-2 cell proliferation induced by miR-7-5p. The hydroxyproline content and mRNA/protein levels of FGFR4 and three HSC activation markers in LX-2 cells were also examined. The miR-7-5p mimic and pcDNA3.1 empty vector treatment greatly increased the hydroxyproline content and marker levels; however, FGFR4 overexpression markedly decreased the hydroxyproline content and marker levels, compared to the NC+Vector group (Figures 5(c)–5(e)). When treated with the miR-7-5p mimic and pcDNA3.1-FGFR4 expression vector, the hydroxyproline content and marker levels in LX-2 cells were markedly reduced compared with the mimic+Vector group, while they were greatly increased compared with the NC+oeFGFR4 group. FGFR4 was significantly downregulated in the mimic+Vector group but markedly increased in the NC+oeFGFR4 group compared to the NC+Vector group (Figures 5(d) and 5(e)). Compared to the mimic+Vector group, FGFR4 was upregulated in the mimic+oeFGFR4 group. Compared to the NC+oeFGFR4 group, FGFR4 was suppressed in the mimic+oeFGFR4 group. These findings suggested that, as a downstream effector regulated by miR-7-5p, FGFR4 overexpression can effectively block miR-7-5p-induced HSC activation.

## 4. Discussion

Our *in silico* analysis implicated that FGFR4 was targeted by miR-7-5p, indicating that miR-7-5p might be involved in liver fibrosis. In pathologic liver tissues, miR-7-5p was markedly increased in severe fibrosis or cirrhosis samples compared to mild fibrosis samples and was more enhanced in cirrhosis samples compared with severe fibrosis samples. On the contrary, FGFR4 expression was significantly downregulated in severe fibrosis or cirrhosis samples compared to mild fibrosis samples, and it was much lower in cirrhosis samples compared with severe fibrosis samples. miR-7-5p was negatively correlated with FGFR4 level in a pathological liver, which was consistent with our *in silico* prediction.

FGF19 is a hormone secreted in the gut and sent to the liver as signals. FGF19 is responsible for bile acid, lipid, and carbohydrate metabolism homeostasis in the liver [23]. Many of FGF19 targets are related to metabolism and proliferation [23]. Several experiments have demonstrated that FGF19 exerts a protective effect against liver fibrosis [24–27]. Binding to FGFR4 is required for FGF19 proliferative function in mouse livers [28, 29]. Using LX-2 cells (a human HSC cell line), we further examined miR-7-5p and FGFR4 function in HSC proliferation and activation. We found that FGFR4 expression was markedly decreased in miR-7-5p-overexpressing LX-2 cells, while it was greatly enhanced in LX-2 cells treated with the miR-7-5p inhibitor, compared with NC and vehicle control. This finding further verified that FGFR4 was negatively modulated by miR-7-5p in the liver. The dual-luciferase assay also verified that miR-7-5p regulated FGFR4 by binding to its 3′-UTR. miR-7 has been reported as a crucial mediator of lipid metabolism in the liver [30]. Downregulation of miR-7 is related to various cancer types [31–33]. In the liver, miR-7 was ascribed a role in tumor suppression [34, 35] and a crucial mediator in the maintenance of lipid homeostasis [30]. Further, we found that LPS treatment promoted LX-2 cell proliferation compared to the vehicle control; however, the miR-7-5p inhibitor and LPS treatment repressed the proliferation capability compared to the NC and LPS treatment groups, indicating that the miR-7-5p inhibitor blocked LX-2 cell proliferation induced by LPS. Besides, overexpression of FGFR4 could rescue the effect of the miR-7-5p inhibitor on LX-2 cell proliferation. Our results indicated that FGFR4 is a putative target gene of miR-7-5p.

Activated HSCs trigger the production of hydroxyproline in the extracellular matrix, and hydroxyproline maintains liver cell integrity and normal function [36]. Therefore, hepatic hydroxyproline content could correctly signify the degree of liver fibrogenesis. Besides, activated HSCs can upregulate *α*-SMA and COL1A1 and generate the hepatic cytokine TGF-*β* [37]. Hence, we measured the hydroxyproline content and expression of COL1A1, TGFB1, and *α*-SMA in LX-2 cells with different treatments to unveil miR-7-5p and FGFR4 function in HSC activation. Our data illustrated that the miR-7-5p inhibitor impaired the LPS-induced upregulation of hydroxyproline content and HSC activation markers in HSCs and FGFR4 overexpression inhibited the miR-7-5p-triggered upregulation of hydroxyproline content and HSC activation markers in HSCs. Under the condition of LPS, FGFR4 was significantly downregulated in the LPS group compared to the vehicle control, while it was markedly increased in the miR-7-5p inhibitor and LPS treatment groups compared to the NC and LPS treatment groups. All these data demonstrated that FGFR4 was downregulated by miR-7-5p.

In summary, our results suggested that miR-7-5p promoted HSC proliferation and activation through downregulating FGFR4. Inhibitory agents targeting miR-7-5p may provide new therapeutic choice for liver fibrosis management.

## Figures and Tables

**Table 1:** Primers used in RT-qPCR.

Gene	Forward	Reverse
miR-7-5p	5′ CGCGTGGAAGACTAGTGATTTT 3′	5′ AGTGCAGGGTCCGAGGTATT 3′
RNU6-1	5′ CTCGCTTCGGCAGCACA 3′	5′ AACGCTTCACGAATTTGCGT 3′
FGFR4	5′ CCCTCGAATAGGCACAGTTAC 3′	5′ GCCTCCAATGCGGTTCTC 3′
TGF-*β*1	5′ CGTGGAGGGGAAATTGAGG 3′	5′ GCCATGAGAAGCAGGAAAGG 3′
*α*-SMA	5′ GACGAAGCACAGAGCAAAAG 3′	5′ ACAGCACCGCCTGGATAG 3′
COL1A1	5′ GAGGCATGTCTGGTTCGG 3′	5′ TGGTAGGTGATGTTCTGGGAG 3′
GAPDH	5′ AATCCCATCACCATCTTC 3′	5′ AGGCTGTTGTCATACTTC 3′

## Data Availability

The data used to support the findings of this study are available from the corresponding author upon request.
